# Haemoglobin and Hematinic Status Before and After Bariatric Surgery over 4 years of Follow-Up

**DOI:** 10.1007/s11695-020-04943-0

**Published:** 2020-09-01

**Authors:** Michael J. Shipton, Nicholas J. Johal, Neel Dutta, Christopher Slater, Zohaib Iqbal, Babur Ahmed, Basil J. Ammori, Siba Senapati, Khurshid Akhtar, Lucinda K. M. Summers, John P. New, Handrean Soran, Safwaan Adam, Akheel A. Syed

**Affiliations:** 1 Salford Royal NHS Foundation Trust, Salford, UK; 2 University of Manchester, Manchester, UK; 3 Manchester University NHS Foundation Trust, Manchester, UK; 4 Manchester Metropolitan University, Manchester, UK; 5 The Christie NHS Foundation Trust, Manchester, UK

**Keywords:** Obesity, Morbid, Gastric bypass, Gastrectomy, Anaemia, Iron deficiency, Vitamin B_12_ deficiency, Folic acid deficiency

## Abstract

**Purpose:**

Bariatric surgery is associated with deficiencies of vitamins and minerals, and patients are routinely advised supplements postoperatively. We studied prevalence of vitamin B_12_, folate and iron deficiencies and anaemia before and after bariatric surgery over 4 years of follow-up.

**Materials and Methods:**

We performed a retrospective cohort analysis of 353 people with obesity, including 257 (72.8%) women, who underwent gastric bypass (252, 71.4%) or sleeve gastrectomy (101, 28.6%) at our National Health Service bariatric centre in Northwest England.

**Results:**

At baseline, mean (standard error) age was 46.0 (0.6) years, body mass index 53.1 (0.4) kg/m^2^, serum vitamin B_12_ 400.2 (16.4) pg/L, folate 7.7 (0.2) μg/L, iron 12.0 (0.3) μmol/L, ferritin 118.3 (8.4) μg/L and haemoglobin 137.9 (0.8) g/L. Frequency of low vitamin B_12_ levels reduced from 7.5% preoperatively to 2.3% at 48 months (*P* < 0.038). Mean folate levels increased from baseline to 48 months by 5.3 μg/L (*P* < 0.001) but frequency of low folate levels increased from 4.7% preoperatively to 10.3% (*P* < 0.048). Ferritin levels increased from baseline to 48 months by 51.3 μg/L (*P* < 0.009). Frequency of low ferritin levels was greater in women (39.1%) than in men (8.9%) at baseline (*P* < 0.001) and throughout the study period. Haemoglobin was low in 4.6% of all patients at baseline with no significant change over the study period.

**Conclusion:**

There were notable rates of haematinic insufficiencies in bariatric surgical candidates preoperatively. Our study lends further support to regular supplementation with vitamin B_12_, folic acid, and iron in people undergoing bariatric surgery.

## Introduction

Obesity management includes lifestyle, dietary and behavioural interventions with or without weight loss pharmacotherapy. Bariatric surgery, however, is the most effective option for achieving substantial, sustained weight loss and improving survival [1, 2]. Globally, gastric bypass (GB) and sleeve gastrectomy (SG) are the two most common types of bariatric surgery [3]. Each limits nutrient absorption by either reducing the stomach volume, as with SG, or a combination of restriction of the gastric capacity and reduction of functional small bowel for nutrient absorption, as with GB. Whilst bariatric surgical procedures are generally safe with low mortality rates and excellent efficacy in weight reduction as well as resolution or improvement of comorbidities such as type 2 diabetes [1, 2, 4–8], both GB and SG are frequently associated with micronutrient deficiencies as a consequence of reduced nutrient intake and absorption [9]. Deficiencies commonly include vitamin B_12_ (cobalamin), folate and iron [10].

### Vitamin B_12_

Cobalamin is an essential co-enzyme in several important enzyme-catalysed reactions that are involved in synthesis of DNA, myelin and fatty acids [11]. The reference nutrient intake (RNI) for vitamin B_12_ is 1.5 μg per day for adults [12]. Dietary sources include meat, fish, eggs and dairy foods and some fortified breakfast cereals. Deficiency can manifest as glossitis; haematological disorders such as megaloblastic anaemia, leukopenia, pancytopenia, thrombocytopenia and thrombocytosis; and neuropsychiatric disorders such as peripheral neuropathy, demyelination of dorsal columns and corticospinal tract; cognitive impairment including dementia-like symptoms and acute psychosis; gait abnormalities, irritability, and loss of proprioception and vibratory sense; and olfactory impairment [13]. Vitamin B_12_ deficiency has been reported to affect 6% of men and women (aged 19–64 years) in the UK [14]. Intrinsic factor and/or gastric acid secretion are essential for the absorption of dietary vitamin B_12_ in the ileum; therefore, in altered physiological or pathological states in which these factors are affected, there is a higher prevalence of vitamin B_12_ deficiency [15]. People who have undergone gastric bypass, sleeve gastrectomy and duodenal switch are therefore at risk of cobalamin deficiency due to surgically distorted anatomy and physiological function. This often manifests after a delay of 2 years post-surgery due to hepatic storage of vitamin B_12_ [16]. It is therefore recommended that patients receive 3-monthly intramuscular hydroxocobalamin injections following bariatric surgery to replenish dwindling stores [17].

### Folate

The RNI for folate is 200 μg per day for adults; women require an extra 100 μg during pregnancy [12]. Women who are planning pregnancy are advised 400 μg of folic acid daily until the 12th week of pregnancy [18]; a higher dose of 5 mg per day is recommended for women who have had bariatric surgery [17, 19]. Dietary sources of folate include green leafy vegetables and fortified cereals and supplements. Folate deficiency after bariatric surgery is less common as it is absorbed throughout the small bowel but daily supplementation is still advised due to limited body stores [17, 20].

### Iron

The RNI for iron is 8.7 mg per day for men and women over 50 years of age and 14.8 mg for women under 50 [12]. Dietary haem iron from animal sources has the most bioavailability; sources include red meat, liver and poultry. Non-haem iron, which is less well absorbed, can be found in vegetables, nuts and eggs [21]. In the UK, bread and flour (other than wholemeal flour) and many breakfast cereals are fortified with iron. Iron absorption can be inhibited or enhanced by certain dietary compounds [22]. Phytates in plant-based foods and polyphenols from tea, coffee, legumes, cereals, fruit and vegetables can inhibit absorption of non-haem iron. Oral calcium inhibits absorption of both haem and non-haem iron. Oral vitamin C may enhance absorption of non-haem iron by reducing ferric ions to more readily absorbable ferrous forms [23].

Iron deficiency resulting in microcytic anaemia is one of the most common nutritional problems following bariatric surgery [20]. This probably relates to the combination of reduced oral intake and reduced gastric acid secretion required for iron absorption, along with bypassing of segments of the small bowel where iron absorption occurs, as in the case of GB [24, 25]. It has also been noted that patients with obesity are more likely to have pre-existing iron deficiency [26]. Consequently, current national guidelines and local guidelines at our centre recommend 45–60 mg of oral elemental iron per day in addition to dietary intake post-bariatric surgery [17, 27].

Previous studies have mostly reported short-term results. We therefore studied longitudinal data on vitamin B_12_, folate, iron, ferritin and haemoglobin levels in a sizable baseline cohort followed up over 4 years after bariatric surgery. Whilst patients with obesity are routinely recommended supplements of iron, multivitamins and hydroxocobalamin following bariatric surgery by learned society guidelines [17, 27], there is limited knowledge of the efficacy of the routinely recommended standard supplements in treating and preventing insufficiencies after bariatric surgery in the longer term.

### Aims

We studied levels of haematinics and haemoglobin in patients undergoing bariatric surgery over 4 years of follow-up at a single centre to identify prevalence of deficiencies pre- and postoperatively.

## Methods

We performed an observational cohort analysis of patients who underwent bariatric surgery between August 2010 and October 2012 at our National Health Service (NHS) university teaching hospital in Greater Manchester, UK. Patients were identified from our bariatric surgery database, and clinical information was collected from electronic patient records. We evaluated all patients who underwent GB (252, 71.4%) or SG (101, 28.6%); 15 patients who underwent other primary bariatric surgical procedures (14 gastric band, 1 biliopancreatic diversion/duodenal switch) were excluded. All 252 GB procedures were performed as laparoscopic Roux-en-Y gastric bypass involving the fashioning of a short 5 cm vertical gastric pouch based on the lesser curvature of the stomach and constructed over a 40-French orogastric tube using staplers. An antecolic antegastric Roux-en-Y gastrojejunostomy was fashioned with the bilioenteric limb measuring 100 cm and the alimentary limb measuring 100–150 cm depending on the patient’s body mass index (BMI). A side-to-side jejunojejunostomy was fashioned using intracorporeal suturing technique, and an end-to-side gastrojejunostomy was constructed using intracorporeally sutured technique over a 40-French orogastric tube. The jejunojejunal and Petersen’s mesenteric defects were routinely closed using nonabsorbable sutures. Laparoscopic SG involved the construction of a vertical gastric sleeve over a 40-French orogastric tube starting 4–6 cm from the pylorus and ending approximately 1 cm lateral to the angle of His using staplers.

All patients were prescribed oral complete micronutrient/multivitamin supplement (Forceval®) including folic acid 400 μg, oral iron (ferrous sulfate 400 mg) and oral combined calcium and vitamin D (elemental calcium 1200 mg and cholecalciferol 800 units) daily, and hydroxocobalamin 1 mg intramuscular injection every 3 months. Scheduled postoperative follow-up appointments were at 6 weeks, 4 months, 12 months and annually thereafter for clinical assessment and blood tests. The data assembled included height, weight and BMI, and serum levels of vitamin B_12_, folate, iron, ferritin, haemoglobin and mean corpuscular volume (MCV) at baseline and 4, 12, 24, 36 and 48 months postoperatively. Low levels of vitamin B_12_ (< 211.0 ng/L), folate (< 4.0 μg/L), iron (< 9 μmol/L in women and < 11 μmol/L in men), haemoglobin (< 115 g/L in women and < 130 g/L in men) and MCV (< 80 fL) were defined as per their respective laboratory recommended reference ranges [28]. Ferritin levels were defined as deficient (< 15 μg/L) or insufficient (< 50 μg/L) [29]. Statistical analysis included descriptive statistics, parametric tests (or non-parametric tests for non-normative data as appropriate) and contingency tables using Fisher’s exact test. *P* < 0.05 was deemed to be statistically significant, and 95% confidence intervals (95% CI) were reported where appropriate. Data were analysed using IBM SPSS 25.0 (Armonk, NY) and GraphPad Prism 7.0 (San Diego, CA).

## Results

We studied longitudinal data on haematinics in 353 patients comprised of 257 (72.8%) women. The mean (standard error of the mean) age was 46.0 (0.56) years and BMI was 53.1 (0.38) kg/m^2^ at baseline; 129 (36.5%) patients had type 2 diabetes; 252 patients (71.4%) had undergone GB and 101 (28.6%) SG; women had significantly lower ferritin, iron and haemoglobin levels compared to men at baseline (Table 1).

**Table 1:** Baseline characteristics of participants by sex and type of primary bariatric surgery

	All (*n* = 353)	Men (*n* = 96)	Women (*n* = 257)	P*	Gastric bypass (*n* = 252)	Sleeve gastrectomy (*n* = 101)	*P*^†^
Age (years)	46.01 (0.56)	45.91 (1.02)	46.04 (0.67)	ns	45.27 (0.65)	47.84 (1.10)	0.046
BMI (kg/m^2^)	53.07 (0.38)	53.34 (0.76)	52.97 (0.45)	ns	53.10 (0.42)	52.98 (0.84)	ns
Vitamin B_12_ (pg/L)	400.22 (16.43)	387.00 (17.48)	405.15 (21.62)	ns	391.07 (17.72)	424.74 (37.54)	ns
Folate (μg/L)	7.68 (0.23)	7.88 (0.40)	7.60 (0.27)	ns	7.35 (0.25)	8.54 (0.47)	0.027
Iron (μmol/L)	11.96 (0.28)	13.57 (0.64)	11.38 (0.29)	< 0.003	12.04 (0.34)	11.74 (0.50)	ns
Ferritin (μg/L)	118.31 (8.43)	175.49 (19.97)	98.96 (8.45)	< 0.001	114.04 (10.63)	127.61 (13.56)	ns
Haemoglobin (g/L)	137.90 (0.75)	149.10 (1.34)	133.68 (0.74)	< 0.001	138.22 (0.83)	137.12 (1.58)	ns
MCV (fL)	87.21 (0.27)	87.44 (0.47)	87.13 (0.33)	ns	87.41 (0.32)	86.73 (0.51)	ns

Values reported as mean (standard error of the mean)

*Men vs. women

†Gastric bypass vs. sleeve gastrectomy

Independent samples *t* test, equal variances not assumed

*ns* non-significant. *BMI* body mass index, *MCV* mean corpuscular volume

### Weight Loss

**Fig. 1:**
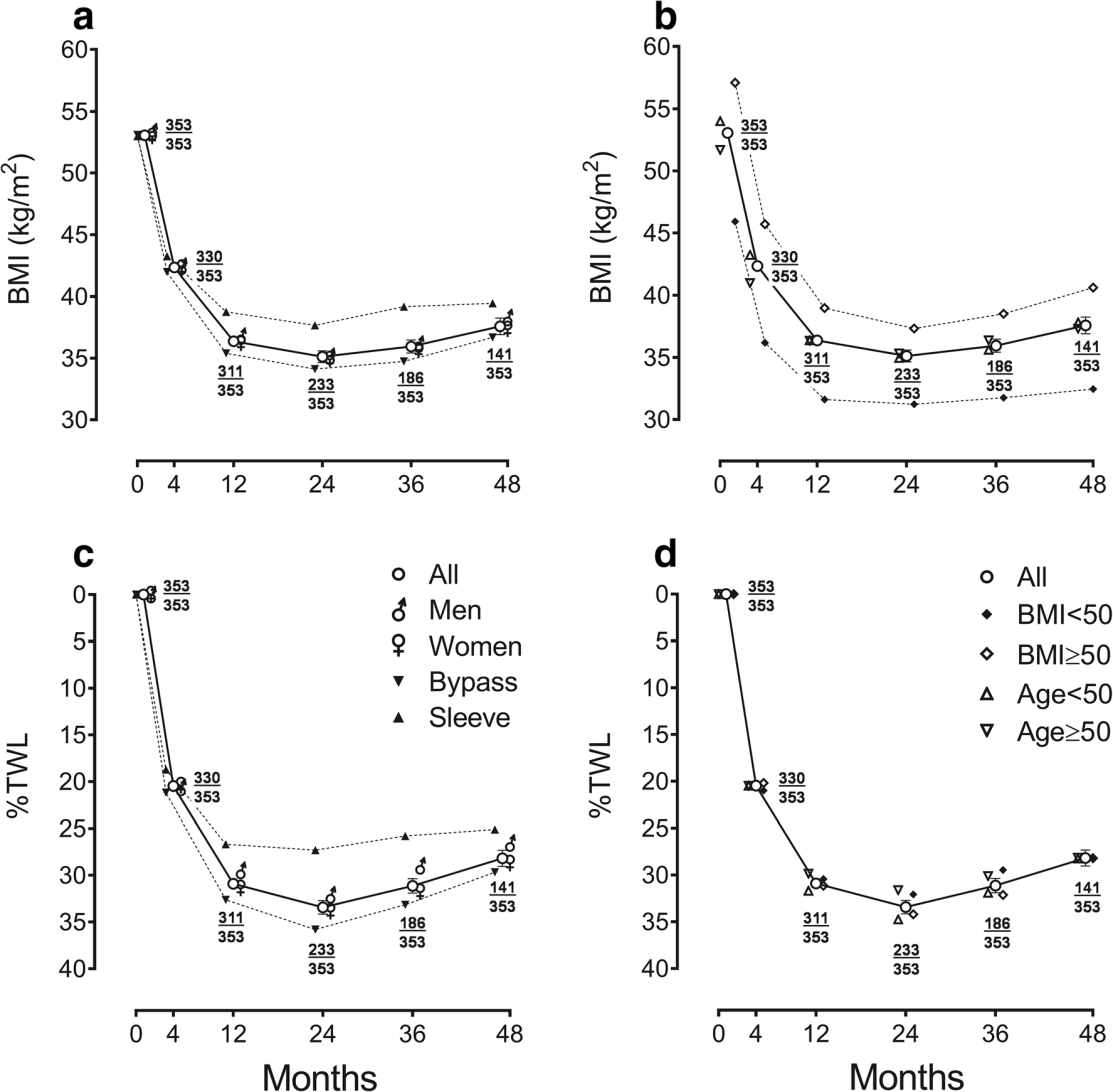
Weight loss outcomes during 4 years of follow-up after bariatric surgery: **a** body mass index (BMI) categorized by sex or type of surgery, **b** BMI categorized by preoperative BMI (< 50 kg/m^2^ or ≥ 50 kg/m^2^) or age (< 50 years or ≥ 50 years), **c** percent total weight loss (%TWL) categorized by sex or type of surgery, and **d** %TWL categorized by preoperative BMI or age. Point values (± error bars) depict means (± standard errors of the means). At each timepoint, fractions depict number of participants contributing data (numerator) over total number of eligible participants (denominator)

There was significant weight loss after bariatric surgery; overall mean difference (95% CI) in BMI from baseline to 48 months was a reduction of 15.2 (14.1 to 16.4) kg/m^2^ (*P* < 0.001). BMI reduction was not significantly different between men and women throughout the period of study but was greater for GB compared to SG with a mean difference (95% CI) of 3.35 (1.63 to 5.07) kg/m^2^ (*P* < 0.001) at 12 months and after (Fig. 1). BMI reduction was not significantly different between younger (preoperative age < 50 years) and older patients but greater in those with lower preoperative BMI (< 50 kg/m^2^) throughout with a mean difference (95% CI) of 8.17 (5.99 to 10.34) kg/m^2^ (*P* < 0.001) at 48 months. Percent total weight loss (%TWL) was greater for GB than SG at 4 months and after with greatest mean difference (95% CI) of 8.50 (5.59 to 11.41)% at 24 months (*P* < 0.001), but there was no difference by sex, age or preoperative BMI.

### Vitamin B_12_

**Fig. 2:**
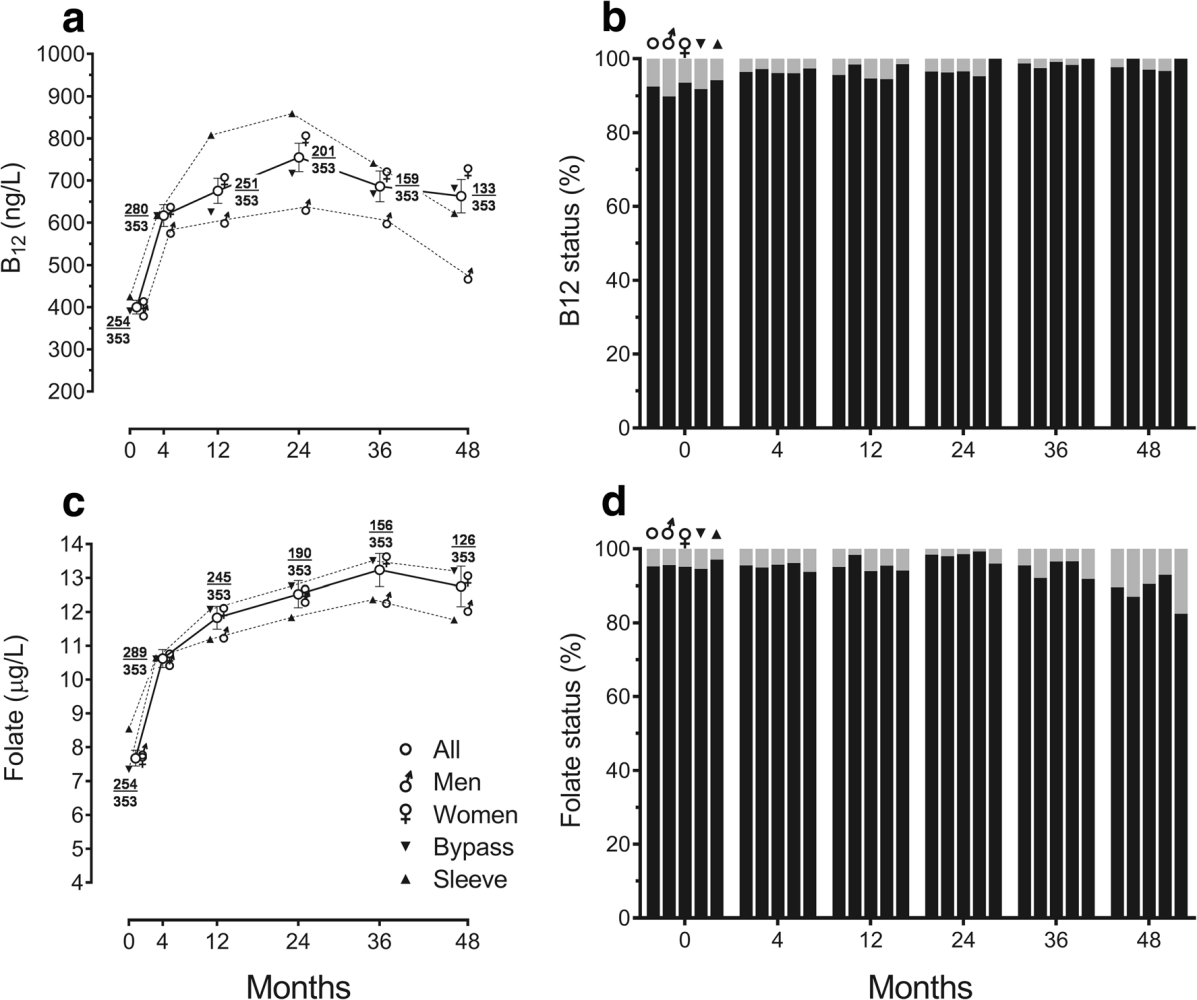
Haematinic levels during 4 years of follow-up after bariatric surgery categorized by sex and type of surgery: **a** vitamin B_12_ levels, **b** proportions of patients with sufficient (black) or low (< 211 ng/L; grey) vitamin B_12_ levels, **c** serum folate levels, and **d** proportions of patients with sufficient (black) or low (< 4.0 μg/L; grey) serum folate levels. In line graphs, points (± error bars) represent means (± standard errors of the means). At each timepoint, fractions depict number of participants contributing data (numerator) over total number of eligible participants (denominator). In column charts, the first column at each timepoint represents all patients, second men, third women, fourth gastric bypass, and fifth sleeve gastrectomy

Low serum vitamin B_12_ levels (< 211 ng/L) were seen in 7.5% of patients overall at baseline compared to 2.3% at 48 months (*P* < 0.038) (Table 2). There were no statistically significant differences in rates of deficiency grouped by sex or type of surgery (Fig. 2). Of 129 people with type 2 diabetes, 104 (80.6%) were taking metformin prior to surgery; median (interquartile range) vitamin B_12_ level in those taking metformin was 349.5 (277.3 to 460.0) ng/L whilst in those not taking metformin was 429.0 (315.0 to 571.0) ng/L (*P* = 0.54).

**Table 2:** Proportion of patients with deficiency in haematinics and haemoglobin and comparison with previous studies

	Baseline	12 months	24 months	36 months	48 months
All patients					
Vitamin B_12_ (%)	7.5	4.4	3.5	1.3	2.3
Folate (%)	4.7	4.9	1.6	4.5	10.3
Serum iron (%)	29.8	15.6	19.3	19.6	27.3
Ferritin (%)	1.1	1.3	4.3	7.1	9.3
Haemoglobin (%)	2.6	4.3	4.7	6.0	7.4
Bypass					
Vitamin B_12_ (%)	8.1 (2.3^a^)	5.5 (6.5^a^)	4.8 (5.4^a^)	1.7 (7.2^a^)	3.3
Folate (%)	5.4 (2.2^a^)	4.5 (1.6^a^)	0.7 (1.8^a^)	3.4 (0.7^a^)	7.0
Iron (%)	28.7 (14.7^b^–15.3^a^)	17.0 (10.5^a^)	17.5 (9.8^a^)	18.6 (10.2^a^)	24.7
Ferritin (%)	1.6	1.2	4.4	7.7	10.3
Haemoglobin (%)	1.6 (12.2^a^)	3.2 (20.9^a^)	5.1 (25.9^a^)	5.7 (23.1^a^)	6.9
Sleeve					
Vitamin B_12_ (%)	5.8 (5.9^d^–9.3^c^)	1.5 (7.8^c^–17.2^d^)	0.0 (1.0^e^)	0.0 (18.0^f^)	0.00
Folate (%)	2.9 (0.0^c^–5.5^d^)	5.9 (13.7^c^–13.8^d^)	4.00	8.1 (22.0^f^)	17.50
Serum iron (%)	32.9 (36.6^b^)	11.9	24.0	22.9	35.1
Ferritin (%)	0.00	1.47	4.08	0.00	7.14
Haemoglobin (%)	4.9	7.3	3.5	7.0	8.7

Values (comparison with previous studies) reported as percentages (%)

^a^Weng et al. [20]

^b^Enani et al. [35]

^c^Antoniewicz et al. [38]

^d^Damms-Machado et al. [32]

^e^Javanainen et al. [40]

^f^Gehrer et al. [37]

Serum vitamin B_12_ levels increased after bariatric surgery (Fig. 2). Mean difference (95% CI) in vitamin B_12_ levels from baseline to 48 months was an increase of 195.1 (84.9 to 305.2) pg/L (*P* < 0.001). Women had higher mean vitamin B_12_ levels compared to men at 24 months (486.9 vs. 436.0 ng/L, *P* < 0.03) and 48 months (482.4 vs. 306.8 ng/L, *P* < 0.001). SG patients had a higher mean vitamin B_12_ level compared to GB at 12 months (807.5 vs. 625.3 ng/L, *P* < 0.013), but not at other timepoints.

### Folate

Low serum folate levels were seen in 4.7% patients overall at baseline compared to 10.3% at 48 months (*P* < 0.048) (Table 2). Rates of folate deficiency increased in SG patients from 2.9% at baseline to 17.5% at 48 months (*P* < 0.012), but there was no statistically significant change in GB patients or men or women (Fig. 2). The frequency of folate deficiency increased in younger patients (preoperative age < 50 years) from 5.7% at baseline to 16.1% at 48 months (*P* < 0.029) and in patients with higher preoperative BMI (≥ 50 kg/m^2^) from 5.7 to 13.9% (*P* < 0.046).

Serum folate levels increased after bariatric surgery (Fig. 2). Mean difference (95% CI) in serum folate levels from baseline to 48 months was an increase of 5.3 (3.8 to 6.8) μg/L (*P* < 0.001). There was no significant difference in mean levels between men and women at any timepoint. SG patients had higher mean serum folate levels compared to GB at baseline (8.54 vs. 7.35 μg/L, *P* < 0.03) (Table 1), but not at postoperative follow-up timepoints.

### Iron and Ferritin

**Fig. 3:**
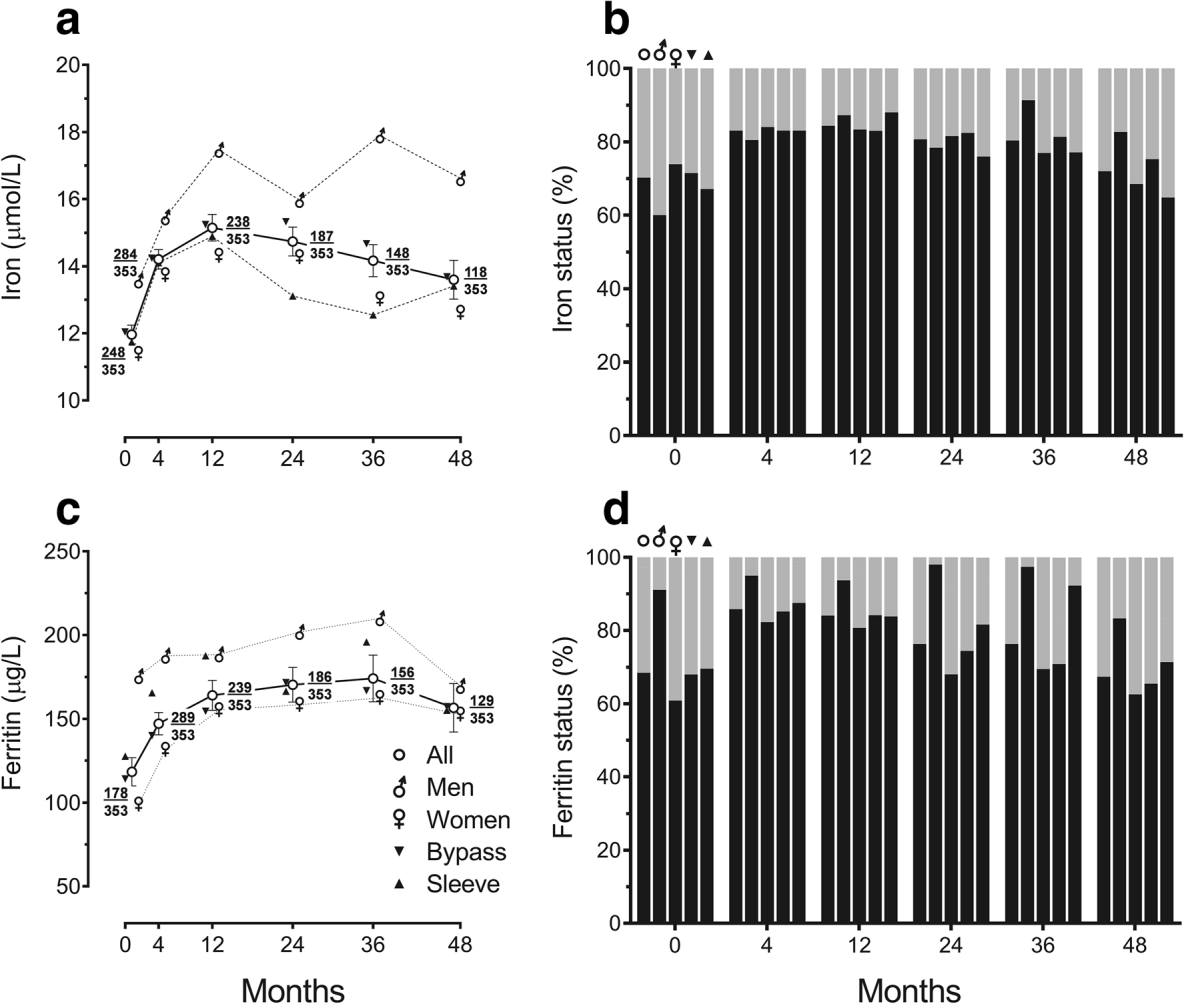
Haematinic levels during 4 years of follow-up after bariatric surgery categorized by sex and type of surgery: **a** serum iron levels, **b** proportions of patients with sufficient (black) or low serum iron levels (< 9 μmol/L in women, < 11 μmol/L in men; grey), **c** serum ferritin levels, and **d** proportions of patients with sufficient (black) or insufficient/low (< 50 μg/L; grey) serum ferritin levels. In line graphs, points (± error bars) represent means (± standard errors of the means). At each timepoint, fractions depict number of participants contributing data (numerator) over total number of eligible participants (denominator). In column charts, first column at each timepoint represents all patients, second men, third women, fourth gastric bypass, and fifth sleeve gastrectomy

Low serum iron levels were seen in 29.8% of all patients with a higher rate in men (40.0%) than women (26.1%; *P* < 0.041) at baseline (Fig. 3), even when comparing to men and women < 50 years (43.2% vs. 29.2%; ns, not significant). At 48 months, the rate of low serum iron reduced to 17.2% in men (*P* < 0.035) but there was no significant change in women at 31.5% overall (ns) and women < 50 years at 37.8% (ns). There was no significant difference in rates of low serum iron between procedures. Serum iron levels increased after bariatric surgery, peaking at 12 months with a slow decline in levels thereafter (Fig. 3). Mean difference (95% CI) in serum iron levels from baseline to 48 months was an increase of 1.99 (0.54 to 3.45) μmol/L (*P* < 0.008). Iron levels were significantly higher in men compared to women at baseline (13.57 vs. 11.38 μmol/L; *P* < 0.003) and 48 months (16.62 vs. 12.62 μmol/L; *P* < 0.006). There was no significant difference between procedures at baseline, but iron levels were higher in GB patients compared to SG patients at 24 months (15.33 vs. 13.11 μmol/L; *P* < 0.013) and 36 months (14.68 vs. 12.54 μmol/L; *P* < 0.036). Older women (preoperative age ≥ 50 years) had higher iron levels compared to younger women at 4 months (14.91 vs. 12.98 μmol/L; *P* < 0.003) and 24 months (16.01 vs. 12.91 μmol/L; *P* < 0.002). Those women who had iron deficiency at baseline had a mean serum iron of 7.01 μmol/L, which increased modestly to 9.61 μmol/L at 48 months (Table 3).

**Table 3:** Trend of haematinic and haemoglobin levels over 4 years of follow-up. Patients were grouped by baseline levels of deficiencies of vitamin B_12_, folate, iron, ferritin and haemoglobin

	Baseline	4 months	12 months	24 months	36 months	48 months
Vitamin B_12_ (pg/L)						
All, not deficient (*n* = 235)	417.98	621.82	671.29	716.60	676.03	646.47
All, deficient (*n* = 19)	180.47	529.93	622.92	763.70	579.90	473.00
Men, not deficient (*n* = 62)	409.42	580.27	598.38	541.57	530.13	395.60
Men, deficient (*n* = 7)	188.43	705.20	751.00	819.25	669.20	440.00
Women, not deficient (*n* = 173)	421.05	636.47	695.01	780.41	718.73	718.14
Women, deficient (*n* = 12)	175.83	442.30	566.00	726.67	490.60	522.50
Folate (μg/L)						
All, not deficient (*n* = 242)	7.89	10.71	11.89	12.45	13.30	12.70
All, deficient (*n* = 12)	3.36	10.33	11.34	13.78	13.44	10.20
Men, not deficient (*n* = 65)	8.06	10.53	11.05	11.61	11.50	10.79
Men, deficient (*n* = 3)	3.80	14.48	14.60	19.80	24.10	16.10
Women, not deficient (*n* = 177)	7.83	10.77	12.16	12.75	13.90	13.35
Women, deficient (*n* = 9)	3.21	08.77	10.88	11.77	10.78	4.30*
Iron (μmol/L)						
All, not deficient (*n* = 175)	13.71	15.07	15.81	15.03	15.06	14.67
All, deficient (*n* = 74)	7.80	11.54	12.75	12.56	11.49	11.46
Men, not deficient (*n* = 39)	16.44	17.50	19.44	16.19	19.63	19.13
Men, deficient (*n* = 26)	9.27	12.57	16.77	14.56	14.88	14.62
Women, not deficient (*n* = 136)	12.93	14.40	14.88	14.71	13.93	13.60
Women, deficient (*n* = 48)	7.01	10.96	10.68	11.37	10.10	9.61
Ferritin (μg/L)						
All, not deficient (*n* = 227)	119.52	156.92	178.33	184.93	194.24	178.20
All, deficient (*n* = 2)	11.50	44.00	31.00	8.00	8.00	5.00
Men, not deficient (*n* = 95)	179.14	189.47	191.03	205.64	215.35	175.24
Men, deficient (*n* = 1)	15.00	46.00	31.00	8.00	8.00	6.00
Women, not deficient (*n* = 132)	99.65	132.65	169.86	170.34	182.03	179.81
Women, deficient (*n* = 1)	8.00	42.00	–	–	8.00	4.00
Haemoglobin (g/L)						
All, not low (*n* = 342)	138.77	140.10	136.71	136.53	136.31	134.33
All, low (*n* = 9)	104.89	117.86	119.17	123.50	117.33	120.00
Men, not low (*n* = 95)	149.43	148.54	146.06	147.75	149.03	145.82
Men, low (*n* = 1)	118.00	129.00	129.00	141.00	132.00	128.00
Women, not low (*n* = 247)	134.67	136.84	133.46	132.45	132.18	130.74
Women, low (*n* = 8)	103.25	116.00	117.20	120.00	114.40	117.33

Values reported as mean

*Data available for one patient only

Serum ferritin levels increased after bariatric surgery (Fig. 3). Mean difference (95% CI) in serum ferritin level from baseline to 48 months was an increase of 51.3 (13.4 to 89.2) μg/L (*P* < 0.009). Ferritin levels were significantly higher in men than in women at baseline (175.5 vs. 99.0 μg/L; *P* < 0.001), but there was no significant difference between procedures. Ferritin levels were low or insufficient in 2.8% and 28.7% of all patients, respectively, at baseline (Fig. 3). Rates of ferritin sufficiency were higher in men than in women at baseline (91.1% vs. 60.9%; *P* < 0.001) and throughout the period of study, but there was no significant difference between procedures.

### Haemoglobin

**Fig. 4:**
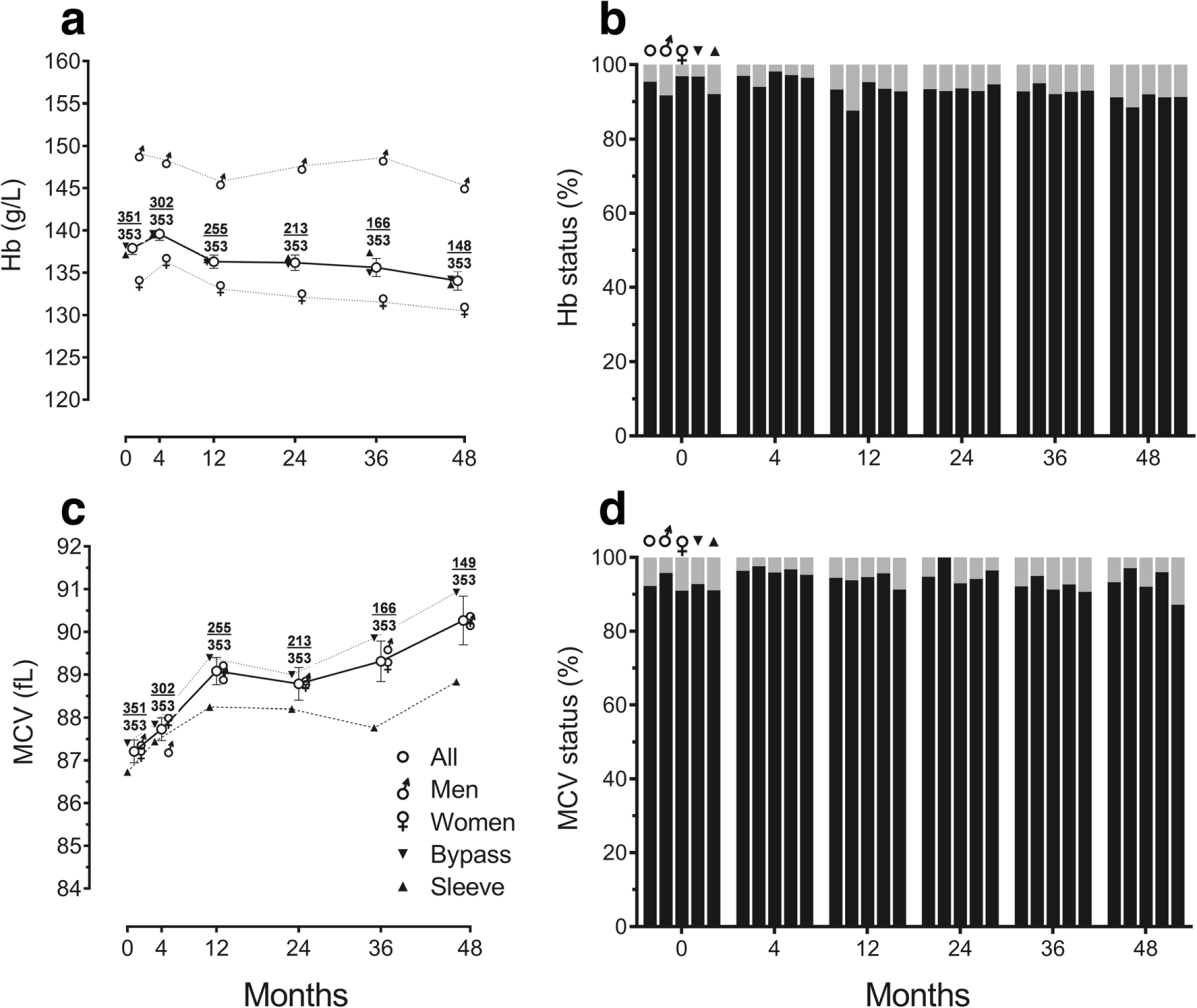
Haematinic levels during 4 years of follow-up after bariatric surgery categorized by sex and type of surgery: **a** haemoglobin (Hb) levels, **b** proportions of patients with sufficient (black) or low (< 115 g/L in women, < 130 g/L in men; grey) haemoglobin levels, **c** mean corpuscular volume (MCV) levels, and **d** proportions of patients with satisfactory (black) or out-of-range (< 80 or > 100 fL; grey) MCV levels. In line graphs, points (± error bars) represent means (± standard errors of the means). At each timepoint, fractions depict number of participants contributing data (numerator) over total number of eligible participants (denominator). In column charts, first column at each timepoint represents all patients, second men, third women, fourth gastric bypass and fifth sleeve gastrectomy

There was no significant change in mean haemoglobin levels overall or within groups throughout the study period (Fig. 4). Men had higher haemoglobin levels compared to women throughout (*P* < 0.001), but there was no significant difference between procedures. Low haemoglobin was seen in 4.6% of all patients at baseline with no significant change over the period of study (Table 2). Rates of anaemia were not significantly different between sexes or procedures. The mean haemoglobin in those women who were anaemic at baseline was 103.25 g/L, and this increased to 117.33 g/L by 48 months (Table 3).

There was no significant difference in mean corpuscular volume (MCV) overall or within groups or between groups throughout the study period (Fig. 4). Low MCV was seen in 7.1% and high MCV in 0.6% of all patients at baseline, with no significant change or difference between groups throughout the study period. Preoperative age and BMI had no significant effect on the frequency of anaemia.

## Discussion

We studied deficiencies of haematinics, namely vitamin B_12_, folate and iron, before and after bariatric surgery over 4 years of follow-up. Very few patients in our cohort had anaemia preoperatively, lower than in the general population or other studies describing 7–22% [20, 30]. However, we report deficiencies of iron in three-tenths, vitamin B_12_ in nearly one-tenth and folate in one-twentieth of patients preoperatively, rates that are higher than in the general population [16, 30, 31], and corroborate previous findings of a prevalence of iron deficiency of 30–40% [26], as well as vitamin B_12_ and folate deficiencies prior to surgery [32, 33].

A higher rate of preoperative low serum iron levels in men was observed in our study, in contrast to previous work which has demonstrated male gender to be protective against iron deficiency [26]. We report an early improvement in iron levels in both men and women, irrespective of type of surgery, but with a similar proportion of patients with low iron levels at 48 months. Women under 50 years had a lower mean serum iron level than those 50 and over, corroborating previous findings of a higher frequency of iron deficiency in menstruating women [34]. There was a significant improvement in iron deficiency in men over this time; however, tendencies towards abrogation of iron deficiency in women and according to each type of surgery remained statistically non-significant. A recent meta-analysis of twenty studies between 2000 and 2015 reported similar absolute rates of iron deficiency when comparing procedures, with a frequency of 22.5% post-GB and 12.4% post-SG, but with an increased risk of iron deficiency after GB but not SG, from a baseline of 14.7% and 36.6%, respectively [35]. This worsening of iron status after GB has been noted elsewhere [20] and is likely due to bypassing of the stomach and duodenum, precluding efficient iron absorption [24]. Iron deficiency may also reflect loss of iron in those women who menstruate, in addition to the population in whom menstruation resumes after bariatric surgery following correction of obesity-related anovulation and insulin resistance [24].

Although mean ferritin levels increased over the course of follow-up in our study, probably related to the supplementation regime and initial good adherence, a higher proportion of patients had insufficiency by 48 months despite an initial rise at 4 and 12 months. This has been noted elsewhere and is thought to correlate with a heightening of the inflammatory state associated with the greater degree of weight loss over the first 6 months postoperatively [36]. In this study by de Cleva et al. [36], iron deficiency anaemia replaced anaemia of chronic disease as the predominant cause of anaemia after GB, attributed to postoperative food aversions, malabsorption, menstruation, and reduction in the obesity-related proinflammatory state.

Obesity is not known as a risk factor for vitamin B_12_ deficiency per se, but may occur in the context of use of metformin and proton pump inhibitors, and poor nutrient intake [16]. Metformin use in our cohort was not associated with vitamin B_12_ deficiency. Vitamin B_12_ deficiency has been reported to be more frequent after GB compared to SG [37–40], probably as a consequence of reduced gastric acid and intrinsic factor production [20], although no significant difference was observed in our study. A meta-analysis of nutrient deficiencies after bariatric surgery found a prevalence of cobalamin deficiency increasing from 2.3% at baseline to 7.2% at 36 months post-GB despite supplementation [20]. This contrasts our findings of a decrease in prevalence of cobalamin deficiency; however, this is unsurprising as previous studies predominantly involved the use of oral multivitamins to provide vitamin B_12_ with only half supplementing with intramuscular cobalamin. Other work has shown that either intramuscular or high-dose oral cobalamin supplementation is required to prevent deficiency [31].

An improvement in the proportion of patients with folate deficiency after GB has been noted [20], which may be attributed to supplementation with multivitamins postoperatively as well as fortification of a host of foodstuffs with folic acid [31]. There is increased risk of folate deficiency post-GB related to decreased gastric acid secretion and reduced absorption due to bypassing of the upper segment of the small intestine [39]. We observed an increased frequency of folate deficiency after SG, but not GB, which has been noted elsewhere [37, 38], although the cause of this remains to be elucidated. Higher preoperative BMI was associated with folate deficiency. This corroborates previous findings [41], and may be related to a high-calorie diet deficient in micronutrients [31].

Weng et al. evaluated nine studies from 2009 to 2014 and found an increase from 12% of patients with anaemia prior to GB to 23% at 36 months, despite supplementation with multivitamins in most reports [20]. We found lower prevalence of anaemia, probably due to our regimen of supplementation with iron tablets containing higher doses of iron. Data regarding the relative risk of anaemia after SG compared to GB remains conflicting. A study of 306,298 patients described a two-fold higher risk of anaemia after GB compared to SG [42], with similar findings reported recently [35]. In contrast, a meta-analysis by Kwon et al. in 2014 did not demonstrate a significant difference in the rates of anaemia between the two types of surgery [43].

A recent study has reported higher rates of anaemia and iron and vitamin B_12_ deficiencies at two and 3 years postoperatively after gastric bypass [44]. Two other studies have reported differing findings regarding the effect of surgery on haematinic status. Misra et al. [45] described no significant difference in rates of anaemia or deficiencies in ferritin, vitamin B_12_ or folate between GB and SG at 3 years’ follow-up, a result echoed in our study. Ledoux et al. [46] similarly reported no effect of type of procedure on vitamin B_12_ deficiency or anaemia, although rates of anaemia increased more substantially after GB; there was a higher frequency of folate deficiency after SG compared to GB.

Clearly there is some heterogeneity, and further work is required to delineate the risk of anaemia attributed to each procedure. Despite an increase in iron and folate deficiency throughout 48 months of follow-up, this was not correlated with a concomitant rise in the frequency of anaemia. Low serum iron levels became more frequent beyond 12 months after surgery. This suggests reduced adherence to recommended supplementation. Haematinic deficiencies remain a risk for development of anaemia, and longer follow-up is required to determine whether these deficiencies would ultimately progress to anaemia.

In an effort to improve the frequency of haematinic deficiencies and consequent anaemia post-bariatric surgery, a multidisciplinary team approach is crucial to encourage patient engagement to continue taking supplements postoperatively, with a particular focus on women of childbearing age [19]. Our practice includes multidisciplinary, patient-focused approach with peer-group support and the improvements in haematinic levels in our study provide evidence of high levels of medication adherence by patients. Additionally, given the high frequency of iron deficiency preoperatively, there may be a case for consideration of providing supplementation even prior to surgery. Obesity itself is associated with reduced serum iron levels, firstly due to poor iron intake and secondly because of hepcidin upregulation as part of the inflammatory milieu, thereby resulting in functional deficiency [47]. Thus, supplementation may not be enough to prevent iron deficiency in either non-surgical candidates or those postoperatively but remains the main treatment option to optimize iron status at present. Parenteral iron is expensive and more difficult to administer, but is effective in cases of refractory iron deficiency anaemia [36].

Studies support the use of a pharmaceutical-grade multivitamin, the use of which resulted in reduction in rates of haematinic deficiencies and anaemia after GB [48, 49]. The multivitamin used in the study by Homan et al. included 350 μg of vitamin B_12_ [48], which may be sufficient to overcome deficiency as 1–5% of vitamin B_12_ is absorbed by an intrinsic factor-independent mechanism [16]. However, other studies demonstrated higher doses of oral hydroxocobalamin are required, with 1000 μg per day proving optimal to manage vitamin B_12_ deficiency after GB [50]. Furthermore, in a study comparing oral and intramuscular cobalamin, there was no significant difference in improvement in vitamin B_12_ levels, suggesting that high-dose oral vitamin B_12_ (1000 μg per day) is sufficient to overcome deficiency [51]. Whether oral vitamin B_12_ can prevent deficiency in a real-world setting remains to be seen.

Limitations of this study include its retrospective design (although data was accumulated prospectively) and data attrition due to patients lost to follow-up. It was beyond the scope of this pragmatic, real-world, retrospective observational study to accurately confirm adherence to recommended post-surgical supplementations. Previous studies have reported adherence to vitamin supplementation post-bariatric surgery may be as low as 30%, and risk factors for poor adherence include male gender, full-time work and attachment anxiety [52]. Poor adherence with iron therapy is well-documented, resulting from various factors including frequent side effects, unavailability of supplements, lack of education and/or support by healthcare professionals, and low demand for treatment due to lack of symptoms of anaemia [53]. In cases where iron deficiency persists despite good adherence, parenteral supplementation is usually required especially in menstruating and pregnant women [54]. It was beyond the scope of this work to ascertain menstruation status in women of childbearing age. We also did not evaluate albumin, copper and zinc levels which can rarely contribute to anaemia.

In summary, we report a high proportion of haematinic deficiencies preoperatively, especially in iron. A strong focus on encouragement of patients to continue taking supplements post-bariatric surgery is imperative, and there may even be an argument for consideration of iron supplementation prior to surgery, especially in pre-menopausal women.
