# Correction to: Haemoglobin and Hematinic Status Before and After Bariatric Surgery Over 4 Years of Follow-Up

**DOI:** 10.1007/s11695-021-05515-6

**Published:** 2021-06-14

**Authors:** Michael J. Shipton, Nicholas J. Johal, Neel Dutta, Christopher Slater, Zohaib Iqbal, Babur Ahmed, Basil J. Ammori, Siba Senapati, Khurshid Akhtar, Lucinda K. M. Summers, John P. New, Handrean Soran, Safwaan Adam, Akheel A. Syed

**Affiliations:** 1 Salford Royal NHS Foundation Trust, Salford, UK; 2 University of Manchester, Manchester, UK; 3 Manchester University NHS Foundation Trust, Manchester, UK; 4 Manchester Metropolitan University, Manchester, UK; 5 The Christie NHS Foundation Trust, Manchester, UK

**Correction to: Obesity Surgery (2021) 31:682–693**

10.1007/s11695-020-04943-0

In the original article, the units for vitamin B_12_ were incorrectly identified in Tables 1 and 3. The correct units are (ng/L). The same error also appeared in the “Results” section of the Abstract and in the “Vitamin B_12_” section of the article text.

